# Co-benefits and trade-offs between environmental impact and diet quality: insights from observational dietary data in a Swedish population

**DOI:** 10.1007/s00394-025-03855-y

**Published:** 2026-01-16

**Authors:** Elinor Hallström, Niclas Håkansson, Ulf Sonesson, Agneta Åkesson, Alicja Wolk

**Affiliations:** 1 RISE Research Institutes of Sweden AB, Gothenburg, Sweden; 2 Institute of Environmental Medicine, Karolinska Institute, Stockholm, Sweden; 3 Department of Surgical Sciences, Uppsala University, Uppsala, Sweden

**Keywords:** Nutrition, Food, Life cycle assessment, Sustainable diets, Dietary guidelines

## Abstract

**Purpose:**

This study characterises food and nutrient intake in self-reported diets with varying environmental impacts and evaluates their adherence to dietary guidelines and nutritional adequacy, exploring co-benefits and trade-offs between environmental impact and diet quality.

**Methods:**

Dietary data were from two population-based cohorts (n = 30 000) of Swedish adults aged 56–70 years. Environmental impact was assessed using an aggregated score based on six environmental indicators. Adherence to dietary guidelines and nutritional adequacy was evaluated against the Nordic Nutrition Recommendations 2023.

**Results:**

In line with the dietary guidelines, diets with a lower environmental impact had *lower* intake of red meat, processed meat, sugar-sweetened beverages, high-fat dairy products, saturated fat, sodium and alcohol, alongside *higher* intake of whole grains, fibre and polyunsaturated fat. Contrary to the dietary guidelines, lower-impact diets had *higher* intake of sweets and snacks and *lower* intake of vegetables, fruits and berries, nuts and seeds, and seafood. While lower-impact diets had a *lower* intake of protein and most vitamins and minerals, adherence to macronutrient recommendations was generally higher, and micronutrient intake was adequate, except for selenium and vitamin D.

**Conclusions:**

This study highlights both co-benefits and trade-offs between environmental and health goals. To prevent negative health effects from diets with lower environmental impact, it is essential to limit foods with low nutritional value (e.g., sweets and snacks) and promote healthy plant-based foods. Potential risks associated with a lower micronutrient intake should be considered, particularly for groups with special requirements, such as children, adolescents, fertile and pregnant women, and the elderly.

**Supplementary Information:**

The online version contains supplementary material available at 10.1007/s00394-025-03855-y.

## Introduction

Sustainable diets should promote health, meet nutritional needs, and remain within planetary boundaries for natural resources and environmental impact [1]. However, diets in affluent regions often fail to meet these criteria, contributing to negative health and environmental outcomes [2, 3]. In Sweden, for instance, dietary risk factors account for an estimated 15% of total mortality [4], and average diets are estimated to exceed per capita planetary boundaries for greenhouse gas (GHG) emissions, cropland use, application of nitrogen and phosphorus, and biodiversity by twofold to more than fourfold [5, 6].

Research suggests that transitioning to more sustainable diets can benefit both human and planetary health [7]. However, trade-offs between health and environmental aspects may occur and require consideration [8, 9]. Concerns include inadequate intake of micronutrients in environmental-adapted diets with fewer animal-based foods [10–12], and the inclusion of nutrient-poor foods with low environmental impact, e.g., sugary snacks [13, 14]. Thus, diets with a lower environmental impact may lead to negative health consequences if nutrition quality is not adequately addressed [15]. Dietary guidelines play a central role in promoting sustainable diets [16]. International authorities such as FAO and WHO have called for integration of health and environmental sustainability in national dietary advice [17]. Such integrated advice only exists in a limited number of countries [18] but was recently implemented in the Nordic Nutrition Recommendations (NNR) 2023, which presents a framework for integrating environmental sustainability in scientific advice for food-based dietary guidelines [19].

Despite growing research on diets’ environmental and health impacts [20], knowledge remains limited, often due to a narrow sustainability perspective on separate aspects. Many studies lack guidance on how dietary patterns can minimize overall environmental impact across multiple indicators and are often based on per capita or modelled diets that fail to capture acceptability aspects and differences between population groups. Moreover, food and nutrient intake differences in diets varying in environmental impact are rarely connected to dietary recommendations needed to assess health implications and support policy. This study aims to address these gaps by identifying self-selected diets varying in environmental impact using a broad environmental perspective, evaluating their link to diet quality, and examine potential co-benefits and trade-offs between the two perspectives. The specific objectives are to:

**(A) Characterise diets with lower and higher environmental impacts**

Differences in food and nutrient intake by quintiles of aggregated environmental impact score, based on six environmental indicators (GHG emissions, cropland use, nitrogen and phosphorus application, consumptive water use, and extinction rate) will be characterised.

**(B) Identify co-benefits and trade-offs between environmental impact and diet quality**

The diet quality of lower and higher environmental impact diets will be evaluated based on adherence to dietary guidelines and nutritional adequacy according to the NNR 2023 [19].

## Methodology

### Study population and assessment of food and nutrient intake

We used dietary data from two population-based cohorts, the Swedish Mammography Cohort (SMC) and Cohort of Swedish Men (COSM) [21]. In short, all women born 1914 to 1948 in two counties were recruited to SMC in 1987–90 (74% response rate). In 1997, COSM was initiated, when all men in two counties, born 1918 to 1952, were recruited, 48,850 responded (49% response rate). A detailed questionnaire and food frequency questionnaire (FFQ) has been completed in both cohorts in 1997 and 2009. For the present study, our study population included the participants who responded to the FFQ in 2009 and at the time were between 56–70 years, to match the given age ranges (51–70 years) for nutritional recommendations against which nutrient intake was compared. Moreover, participants with inadequately reported intake (energy intake outside three standard deviations of log-transformed mean) were excluded. In total, 14,362 women and 15,308 men were included. Both cohorts were representative of the Swedish population in the age group of 56–70 years in terms of similar age distribution, attained education and prevalence of overweight compared to Statistics Sweden 2009.

Dietary information was derived from a 132-item food-frequency questionnaire (FFQ) completed in 2009 (available at www.simpler4health.se). Participants indicated their average consumption of each food item over the past year, choosing from eight predefined frequency categories, ranging from never/seldom to three or more times per day. The individual total energy intake per day was estimated by multiplying intake (frequencies derived from the FFQ multiplied by sex- and age-specific portions sizes) by the energy content of foods and beverages. Food intake was assessed as the quantity eaten, i.e., in cooked weight after weight changes through cooking, and composite dishes were split into individual ingredients based on recipes. Sodium intake did not encompass variations in individual salting habits (standard recipes). Vegetable oils and spreads were included in the FFQ as qualitative questions only, and their contribution to nutrient intake was estimated using standard recipes, which did not allow for quantification of individual intake.

Nutrient and total energy intake were derived from the FFQ using food composition values obtained from the Swedish Food Agency database. Nutrient intake from supplements was not accounted for. The reproducibility and validity of the FFQs were assessed for food and nutrient intake by comparison with multiple 24-h recall interviews and/or diet records [21].

### Assessment of dietary environmental impact

The environmental impact of food items was calculated based on life cycle assessment (LCA) data for six indicators: GHG emissions (kg of CO_2_e), cropland use (m^2^), nitrogen (N) application (kg of N), phosphorus (P) application (kg of P), consumptive water use (m^3^) and extinction rate (E/MSY = extinctions per million species-years). System boundaries included primary production, processing, packaging, international transportation and edible food loss and waste along the food chain (including consumer waste). LCA data from Moberg et al. [5] and further modified by Hallström et al. [6, 22] were used for the environmental assessement.

Each participant’s dietary environmental impact was estimated by matching self-reported diets with LCA data. Impact was assessed per 1000 kcal of food consumed and categorised into quintiles based on the diet’s impact on each of the six environmental indicators. The aggregated environmental score was calculated in line with approaches previously suggested by Fresán et al. [23, 24] and Seconda et al. [25]. In short, a value of 1 to 5 was assigned to each of the six environmental indicators using the study population quintile values as cut-offs, where one point was assigned to study participants in the first quintile, two points to participants in the second quintile and so on. The points for each environmental indicator were added and resulted in an aggregated environmental score ranging from 6 (lowest impact) to 30 (highest impact). New quintiles were then constructed from these scores (Online Resource Table S1), representing participants with the lowest (Q1) and highest (Q5) aggregated environmental impact from the diet (more details in Online Resource). Results were reported as the mean scores for men and women separately.

### Characterisation of diets varying in environmental impact—food and nutrient intake

Food and nutrient intakes per 1000 kcal were assessed across quintiles of aggregated environmental impact (Q1-Q5). Intake of 15 aggregated food groups, 42 sub-groups of food (Online Resource Table S2), and 34 nutrients were reported as the mean intake and standard deviation for men and women separately. Linear regression analyses were used to examine associations between quintiles of aggregated dietary environmental impact and intake of specific foods and nutrients. A p-value of < 0.05 was considered significant.

Differences in the energy standardised intake (per 1000 kcal) between the highest and lowest quintiles of dietary environmental impact were deemed as large if they exceeded 50%, moderate for 20–50%, and small for less than 20%. For nutrient intake, the corresponding cut-offs were > 20%, 10–20% and < 10% for large, moderate and small differences, respectively.

### Quality of diets varying in environmental impact

Diet quality across quintiles of aggregated environmental impact was assessed based on adherence to dietary guidelines and nutritional adequacy.

Adherence to dietary guidelines was assessed by comparing mean food intake in men and women to the NNR 2023 food-based dietary guidelines for adults [19]. Mean food and nutrient intake were assessed per 1984 kcal (8.3 MJ) for women and 2462 kcal (10.3 MJ) for men, based on gender-specific reference values for daily energy intake of adults aged 51–70 calculated using Nordic reference weights and a physical activity level of 1.6. Adherence to dietary guidelines was assessed for intake of red meat; milk and dairy; fish and seafood; vegetables; fruits and berries; nuts; and whole grains, for which quantitative guidelines are proposed in the NNR 2023. Food intake levels were assessed accordance to these guidelines. For example, vegetable intake excludes potatoes and legumes; and meat and fish intake refers to cooked amounts, including the entire content of e.g., sausages. Milk and dairy intake includes milk, yoghurt and cheese but not butter, cream and crème fraiche, and is estimated in dairy equivalents. Methods for calculating dairy equivalents vary by country [26]. In this study, cheese intake was multiplied by a factor of 6 when calculating dairy equivalents, consistent with other European countries (median value: 6.1) [26] and the NNR range of 5–10 [19]. The factor corresponds to the ratio (6.6) of the calcium content of hard cheese (33% fat) and milk (1.5% fat) based on data from to the Swedish Food Agency Database. Intake of milk and yoghurt was calculated based on original intake levels without conversion.

Nutritional adequacy was assessed by comparing mean nutrient intake in men and in women to gender- and age-specific reference values from the NNR 2023 [19]. Macronutrient intake was evaluated against recommended intake ranges and targets for dietary planning, reported as a percentage of total contribution of energy (E%) in adults. Micronutrient intake was evaluated against average requirements (AR) or provisional AR if AR values were missing, and cross-checked against upper intake levels of vitamins and minerals for adults. Nutrients assessed included those with available intake data and reference values specified by the NNR 2023 [19].

## Results

### Characterisation of diets varying in environmental impact

To identify the characteristics of diets varying in environmental impact, food and nutrient intake was assessed across quintiles of aggregated environmental impact. Quintile cut-off values for environmental impact in men and in women are shown in Online Resource Table S3.

#### Food intake

The main findings for food intake are summarised in Table 1, where the arrows describe the association between intake of specific food groups and dietary environmental impact, highlighting the magnitude of the difference in food intake between groups with the highest and lowest dietary environmental impacts.

**Table 1:**
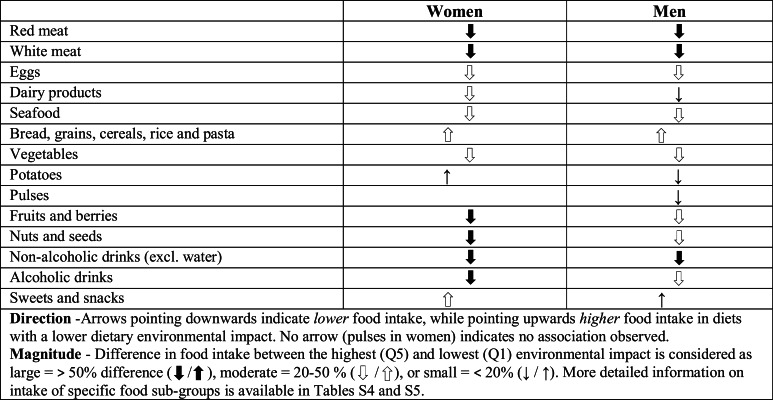
Association between food intake (g/1000 kcal) and environmental impact based on age-adjusted linear regression (p < 0.05). Arrows represent direction and magnitude of differences between the highest and lowest environmental impact

A lower dietary environmental impact was associated with *higher* intake levels of bread, grains, cereals and pasta; sweets and snacks; and potatoes (women), and *lower* intake of red meat; white meat; eggs; dairy products; seafood; vegetables; potatoes (men); pulses (men); fruits and berries; nuts and seeds; non-alcoholic drinks; and alcoholic drinks. Detailed mean food intakes in grams per 1000 kcal by all quintiles of environmental impact are presented for women in Online Resource Table S4 and for men in Online Resource Table S5.

**Fig. 1:**
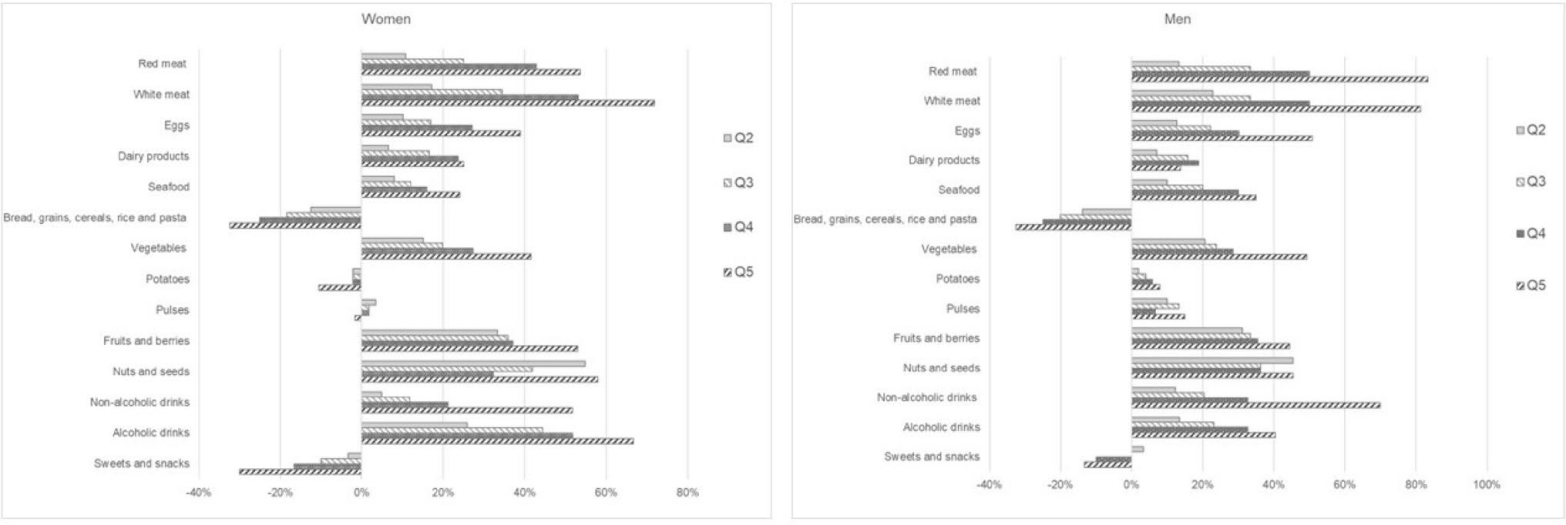
Difference in mean food intake levels between quintiles of dietary environmental impact, in women and men, in relation to Q1 (lowest impact = 0%)

Large intake differences (> 50%) between the highest (Q5) and lowest (Q1) quintiles of dietary environmental impact were observed for red meat; white meat; non-alcoholic drinks; nuts and seeds (women); alcoholic drinks (women); and fruits and berries (women) (Fig. 1). Moderate differences (20–50%) were found for intake levels of eggs; vegetables; fruits and berries (men); nuts and seeds (men); alcoholic drinks (men); seafood; bread, grains, cereals, rice and pasta; dairy products; (women); and sweets and snacks (women). Small intake differences (< 20%) were observed for other aggregated food groups.

#### Nutrient intake

The main findings for nutrient intake are summarised in Table 2, where the arrows describe the association between intake of specific nutrients and dietary environmental impact, highlighting the magnitude of difference in nutrient intake between groups with the highest and lowest dietary environmental impacts.

**Table 2:**
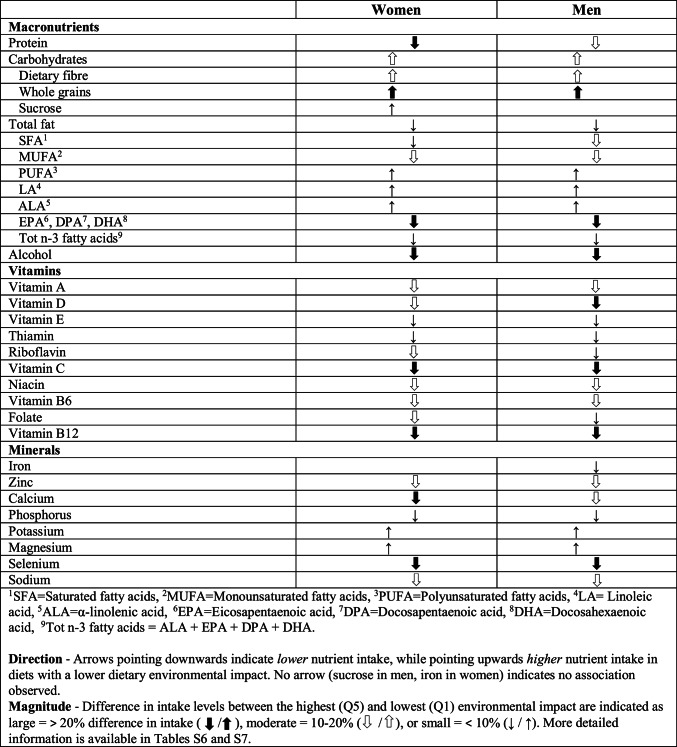
Association between specific nutrient intake per 1000 kcal and environmental impact based on age-adjusted linear regression (p < 0.05). Arrows indicate direction and magnitude of differences between the highest and lowest environmental impact

#### Macronutrients and whole grains

A lower dietary environmental impact was associated with *higher* intake of total carbohydrates, dietary fibre, whole grains, sucrose (women), polyunsaturated fatty acids, linoleic acid and α-linolenic acid, and *lower* intake of protein, total fat, saturated fatty acids, monounsaturated fatty acids, EPA, DPA, DHA, total n-3 fatty acids and alcohol.

**Fig. 2:**
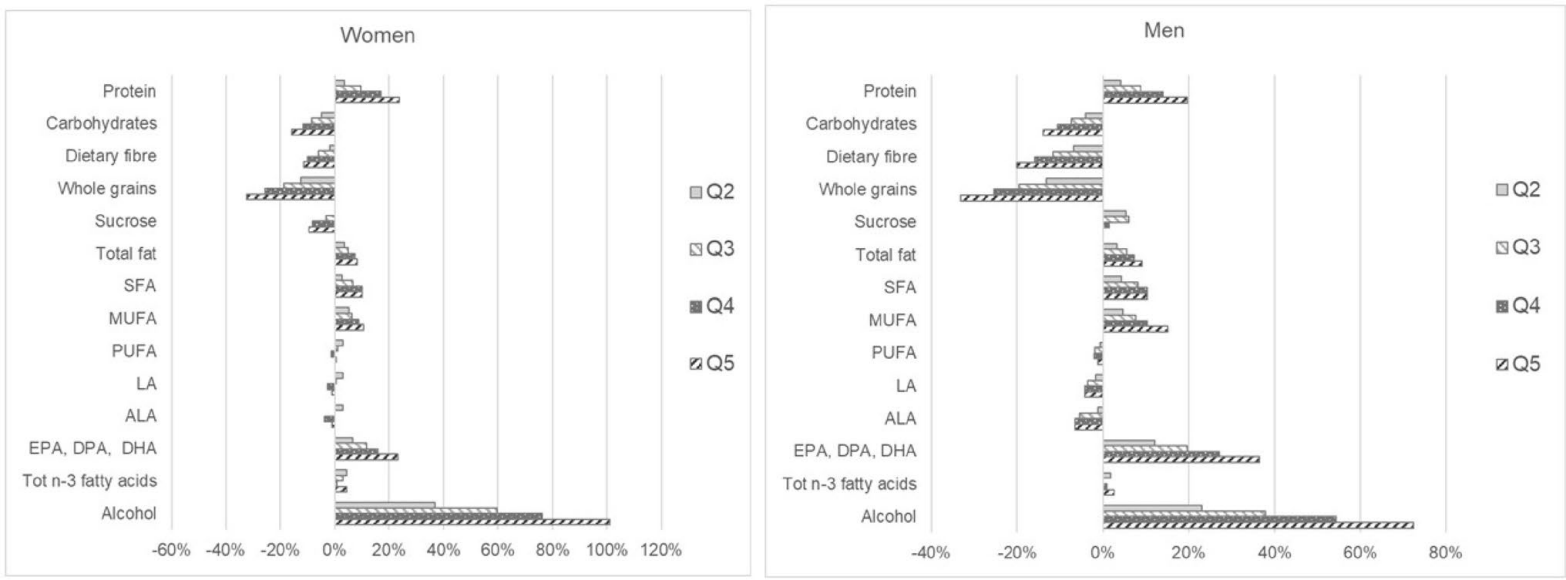
Difference in mean macronutrient intake levels between quintiles of dietary environmental impact, in women and men, in relation to Q1 (lowest impact = 0%)

Large intake differences (> 20%) between the highest (Q5) and lowest (Q1) quintiles of dietary environmental impact were observed for alcohol; whole grains; protein; EPA, DPA and DHA (Fig. 2). Moderate differences (10–20%) were found for intake levels of carbohydrates; fibre; monounsaturated fatty acids and saturated fatty acids. Small intake differences (< 10%) were observed for other macronutrients.

#### Micronutrients

A lower dietary environmental impact was associated with *lower* intake for most vitamins and minerals, except for potassium and magnesium where lower dietary environmental impact was associated with *higher* intake levels.

**Fig. 3:**
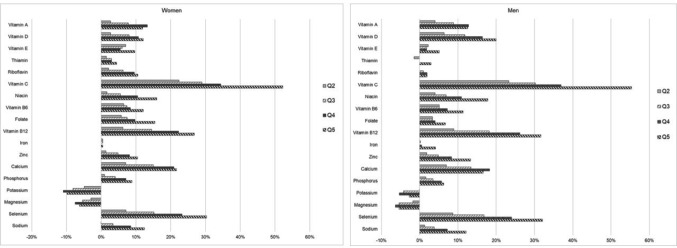
Difference in mean micronutrient intake levels between quintiles of dietary environmental impact, in women and men, in relation to Q1 (lowest impact = 0%)

For micronutrients, large intake differences (> 20%) between the highest (Q5) and lowest (Q1) quintiles of dietary environmental impact were observed for vitamin C; vitamin B12; selenium; calcium (women) and vitamin D (men) (Fig. 3). Moderate differences (10–20%) were observed for intake levels of niacin; calcium (men); folate (women); vitamin A; vitamin D (women); sodium; zinc; riboflavin (women) and vitamin B6. Small intake differences (< 10%) were observed for other micronutrients. Estimates of mean nutrient intakes per 1000 kcal by all quintiles of environmental impact are presented for women in Online Resource Table S6 and for men in Online Resource Table S7.

### Quality of diets varying in environmental impact

#### Adherence to dietary guidelines

Mean daily dietary intake by quintiles of environmental impact is presented for women and men in Table 3. Red meat intake exceeded upper recommended intake in all quintiles in women and men, with the lowest intake (closer to recommendations) in Q1. In men, the intake of processed red meat alone exceeded the upper recommended level of red meat. Milk and dairy intake exceeded upper recommended intake in all quintiles in women and men, with lowest intake (closer to recommendations) in Q1. Fish and seafood intake met dietary guidelines in all quintiles in women and men (intake in Q5 was slightly higher than the recommended range in men), while fatty fish intake was slightly lower than recommended (highest in Q5). Intake of vegetables, fruits and berries was below dietary guidelines across all quintiles in women and men, except for in Q5 in women. Intake was highest (closer to recommendations) in Q5 in both women and men. Intake of nuts was below dietary guidelines in all quintiles in women and men, with highest intake (closer to recommendations) in Q5. Whole grain intake met dietary guidelines in Q1 in women and men, and in Q2 in women, while intake in quintiles of higher dietary environmental impact was lower than recommended.

**Table 3:** Mean food intake in grams per day^1^ by 1st, 3rd and 5th quintiles of dietary environmental impact, in reference to dietary guidelines in NNR 2023^2^

Daily intake	Quintiles of dietary environmental impact						Nordic nutrition recommendations^2^
	Q1		Q3		Q5		
	Women	Men	Women	Men	Women	Men	
Red meat, g/d Processed red meat	**56** **37**	**73** **44**	**70** **44**	**98** **55**	**85** **49**	**135** **67**	Max 50 g/d^3^ As low as possible
Milk and dairy, ml or g/d (dairy Eq. ^4^)	**643**	**777**	**796**	**916**	**1159**	**978**	350–500 ml or g/d^4^
Fish and seafood, g/d Fatty fish	49 **21**	49 **21**	56 **24**	59 **24**	61 **26**	**67** **27**	43–64 g/d^5^ At least 29 g/d
Vegetables, fruits and berries, g/d^6^	**371**	**266**	**471**	**340**	543	**391**	At least 500–800 g/d
Nuts, g/d	**3.4**	**1.2**	**4.8**	**1.6**	**5.4**	**1.8**	20-30 g/d
Whole grains, g/d	100	131	**81**	106	**68**	**88**	At least 90 g/d

Q5 represent diets with the highest environmental impact. Figures in bold indicate intake levels not in line with dietary guidelines

^1^Expressed at the reference energy intake of 8.3 MJ/d for women and 10.3 MJ/d for men [14]. ^2^ Food-based dietary guidelines according to overall science advice, converted to intake per day [14]. ^3^ Max. 350 g red meat per week (ready-to-eat weight). ^4^ Intake levels and dietary guidelines for milk and dairy are calculated as the intake of milk, yoghurt and cheese (excluding butter, cream and crème fraiche), intake of cheese is multiplied by a factor of six to be comparable with dietary guidelines specified in dairy equivalents. ^5^300–450 g seafood per week (ready-to-eat weight, at least 200 g per week should be fatty fish). ^6^Intake levels and dietary guidelines for vegetables, fruits and berries exclude potatoes and pulses

#### Nutritional adequacy

Mean nutrient intake per day by quintiles of dietary environmental impact is presented for women and men in Tables 4 and 5.

**Table 4:** Mean intake of macronutrients per day^1^ by 1st, 3rd and 5th quintiles of dietary environmental impact, in reference to nutrition recommendations in NNR 2023

Daily intake	Quintiles of dietary environmental impact						Nordic nutrition recommendations^7^
	Q1		Q3		Q5		
Macronutrients	Women	Men	Women	Men	Women	Men	
Protein, E%	17	16	18	18	20	19	10–20 (15) E%^8^
Carbohydrates, E%	48	49	**44**	45	**40**	**42**	45–60 (52–53) E%
Dietary fibre, g/MJ	3.6	3.5	3.4	3.1	3.2	**2.8**	≥ 3.0 g/MJ
Total fat, E%	31	29	32	31	33	32	25–40 (32–33) E%
Saturated fatty acids, E%	**13**	**12**	**13**	**13**	**14**	**14**	< 10 E%
MUFA^2^, E%	10	10	11	10	11	11	10–20 E%
PUFA^3^, E%	5.2	**4.8**	5.3	**4.7**	5.2	**4.8**	5–10 E%
LA^4^ + ALA^5^, E%	4.7	4.4	4.7	4.2	4.7	4.2	≥ 3 E%
ALA^5^, E%	0.9	0.8	0.9	0.8	0.9	0.8	≥ 0.5 E%
Total n-3 fatty acids^6^, E%	1.2	1.1	1.2	1.1	1.3	1.1	≥ 1 E%
Alcohol, E%	**1.4**	**2.1**	**2.2**	**2.9**	**2.8**	**3.7**	Avoid or limit

Q5 represent diets with highest environmental impact. Figures in bold indicate intake levels not in line with nutrition recommendations

^1^Expressed at the reference energy intake of 8.3 MJ/d for women and 10.3 MJ/d for men [14]. ^2^MUFA = Monounsaturated fatty acids. ^3^PUFA = Polyunsaturated fatty acids. ^4^LA = Linoleic acid. ^5^ALA = α-linolenic acid**. **^**6**^Total intake of ALA, EPA, DPA and DHA. ^7^Daily recommended intake levels (target for dietary planning) [14]. ^8^Protein should provide 10–20 E% in the adult population and 15–20 E% in the elderly (≥ 65 years)

**Table 5:** Mean intake of micronutrients per day^1^ by 1st, 3rd and 5th quintiles of dietary environmental impact, in reference to average daily requirements in NNR 2023

	Quintiles of dietary environmental impact						Nordic Nutrition Recommendations^2^		
Daily intake	Q1		Q3		Q5		Women		Men
	Women	Men	Women	Men	Women	Men			
*Vitamins*									
Vitamin A, RE/d	1269	1412	1369	1540	1420	1590	530		610
Vitamin D, µg/d	**7.4**	8.1	8.0	9.1	8.3	9.8	7.5		7.5
Vitamin E, mg/d	10	11	10	11	11	12	8		9
Thiamin, mg/d	1.3	1.6	1.4	1.6	1.4	1.7	0.6^3^		0.7^3^
Riboflavin, mg/d	1.9	2.6	2.0	2.6	2.1	2.6	1.3		1.3
Vitamin C, mg/d	102	92	132	120	156	143	75		90
Niacin, NE/d	36	43	38	46	42	51	11^4^		13^4^
Vitamin B6, mg/d	2.1	2.4	2.3	2.5	2.4	2.7	1.3		1.5
Folate, µg/d	346	366	373	380	401	391	250		250
Vitamin B12, µg/d	7.0	8.0	8.0	9.5	8.8	11	3.2		3.2
*Minerals*									
Iron, mg/d	13	15	13	15	13	16	6		7
Zinc, mg/d	12	15	13	15	13	17	7.9		10.4
Calcium, g/d	1.1	1.3	1.3	1.5	1.3	1.5	0.75		0.75
Phosphorus, g/d	1.7	2.0	1.7	2.1	1.8	2.2	0.42		0.42
Potassium, g/d	5.0	5.3	4.6	5.1	4.5	5.1	2.8		2.8
Magnesium, g/d	0.48	0.55	0.45	0.52	0.45	0.52	0.24	0.28	
Selenium, µg/d	**42**	**48**	**48**	**56**	**55**	**64**	60	70	
Sodium, g/d	**2.8**	**3.4**	**2.9**	**3.6**	**3.1**	**3.9**	< 2.3^5^	< 2.3^5^	

Q5 represent diets with the highest environmental impact. Figures in bold indicate intake levels not in line with nutrition recommendations

^1^Expressed at the reference energy intake of 8.3 MJ/d in women and 10.3 MJ/d in men [14]. ^2^Average daily requirements (AR) of vitamins and minerals for women/men, 51–70 years [14], except for sodium. ^3^Recalculated AR per day based on original AR of 0.07 mg/MJ. ^4^Recalculated AR per day based on original AR of 1.3 NE/MJ. ^5^Daily intake of sodium is compared to the chronic disease risk reduction intake value for sodium since a reference value for AR is not available

For macronutrients (Table 4), the carbohydrate intake was below the recommended intake in Q5 in women and men, and in Q3 in women. Fibre intake was below the recommended intake in Q5 in men. Saturated fat intake exceeded maximum recommended intake in all quintiles in both women and men, with lowest intake (closer to recommendations) in Q1. Polyunsaturated fat intake was below the recommended intake in all quintiles for men. Alcohol intake ranged from 1.4–2.8 E% in women and 2.1–3.7 E% in men across quintiles, exceeding the recommendation to avoid or limit alcohol intake to very small amounts, with the lowest intake (closer to recommendations) found in Q1. For all other macronutrients, intake met nutrition recommendations across all quintiles in both women and men.

For micronutrients (Table 5), the intake of vitamin D was below AR in Q1 in women. Selenium intake was below AR in all quintiles in both women and men, with the highest intake (closer to AR) in Q5. Sodium intake exceeded maximum levels recommended in all quintiles in both women and men. Intake of all other micronutrients were above AR (but below upper intake levels) across all quintiles in both women and men.

## Discussion

This study identifies key characteristics of food and nutrient intake in self-reported diets with varying impact across multiple environmental indicators, shedding light on dietary changes that can reduce environmental impact while remaining realistic and acceptable for the Swedish population. The study also evaluates how these lower and higher environmental impact dietary patterns align with food and nutrition recommendations, highlighting potential co-benefits and trade-offs between diet quality and environmental impact, which are important to consider in future policies and interventions.

### Characteristics of diets varying in environmental impact and implications for diet quality

Self-reported diets with a lower environmental impact were characterised by higher intake of bread, grains, cereals and pasta; sweets and snacks; and potatoes in women, and lower intake of the remaining food groups including animal-based foods (e.g., red meat, white meat, dairy products, seafood, eggs), plant-based foods (e.g., vegetables, fruits and berries, nuts and seeds) and beverages (non-alcoholic drinks and alcoholic drinks). Foods with the largest intake variations between high and low environmental impact (> 50%) were red meat, white meat, non-alcoholic drinks, nuts and seeds, alcoholic drinks, and fruits and berries. Targeting these food groups could be crucial for reducing environmental impact.

The characteristics of diets with lower environmental impact are partly in line with Nordic dietary guidelines [19], recommending a *limited* intake of red meat, processed meat, sugar-sweetened beverages, alcohol and high-fat dairy products, and *increased* intake of whole-grain products and potatoes compared to current levels. Only the lowest environmental impact group (Q1) met recommended intake of whole grains in both sexes and this group was also closest to dietary recommendations for red meat, processed meat, and milk and dairy. However, potential trade-offs were also found between the characteristics of lower dietary environmental impact and dietary recommendations, including higher intake of sweets and snacks and lower intake of vegetables, fruits and berries, nuts and seeds, and seafood. In fact, only the highest environmental impact group (Q5) met recommended intake levels for vegetables and for fruits and berries in men, and this group was also closest to the recommended levels in women. This indicates that characteristics of self-reported diets with a lower environmental impact may have both positive and negative implications on diet quality.

Although increased plant-based intake is recommended as a key strategy for healthier, low environmental impact diets [1, 19], this study found that diets with lower impact were associated with lower intake of most plant-based food groups, except for cereals, grains and breads, and sweets and snacks. Previous research in Sweden demonstrated that shifting to more plant-based diets can reduce environmental impact while meeting most nutritional recommendations [27]. However, higher intake of sugar and sweets has also been noted in low climate impact Swedish diets [14, 28]. In Canada, self-selected low climate impact diets also showed higher intake of cereals, grains and breads and lower intake of vegetables and fruits [29]. These findings highlight the need for more research on the role of different plant-based foods in healthy, low impact diets across multiple environmental indicators.

### Nutritional benefits and risk of diets with a lower environmental impact

Diets with a lower environmental impact were associated with several nutritional benefits including higher intake of fibre and polyunsaturated fat, and lower intake of saturated fat, sodium, and alcohol. These diets also had higher adherence to nutrition recommendations for carbohydrates, fibre, saturated fat, sodium and alcohol – nutrients for which adherence to recommendations is low in the Swedish population. For example, 70–80% of Swedish adults eat less fibre and more saturated fat and sodium than recommended [30]. High intake of sodium and low intake of fibre and polyunsaturated fat are, moreover, ranked as leading dietary risk factors to disease burden in Sweden [31], and may thus provide a win–win opportunity for positive health effects and lower environmental impact.

On the other hand, lower dietary environmental impact was associated with potential nutritional risks including higher intake of sucrose and lower intake of most vitamins and minerals. However, these differences in nutrient intake had little effect on adherence to nutrition recommendations. In fact, the lowest environmental impact group (Q1) met the AR for all vitamins and minerals, except for selenium and vitamin D in women. For all other nutrients, the intake even met recommended intake (RI) levels. National dietary surveys in Sweden indicate that most micronutrients are adequately consumed, except for vitamin D, iron (in women), folate, selenium and potassium [30, 32]. The observed association between lower environmental impact and lower intake of vitamin D, folate and selenium may therefore be of special concern in the Swedish population. In this study, the mean intake of vitamin D among women in Q1 was below AR, whereas intake in Q3 and Q5 reached AR but not RI. Obtaining adequate levels of vitamin D is a particular concern in the Nordic countries due to limited sun exposure in the winter months [33]. In Sweden, seafood and dairy products are main dietary sources of vitamin D [30], so reducing intake of these food groups requires attention to ensure adequate vitamin D levels. Furthermore, the mean intake of selenium in this study was below AR across all quintiles of dietary environmental impact and only reached 70% of AR in Q1. Low levels of selenium are common in Europe [34], also in the Nordic countries where the concentration of selenium is low in locally produced foods due to a low soil selenium content [19]. As seafood, meat and dairy products are main sources of selenium [30] polices for lower intake of animal-based foods should address selenium intake, especially if combined with advice on locally produced food in the Nordic context.

Lower intake of protein, omega-3 fatty acids and uptake of minerals are other nutritional aspects raised as potential trade-offs of diets with a lower environmental impact. This study confirmed an association between lower dietary environmental impact and lower protein intake, with a more than 20% difference in intake between the highest and lowest impact groups. However, protein intake in Q1 still met recommended levels. Protein deficiency is uncommon in Sweden [35], and mean protein intake varies between 15 and 19 E% in the Nordic countries [36]. Adopting diets with lower environmental impact may also affect protein quality, as plant protein generally has lower digestibility and bioavailability compared to animal protein and requires a combination of dietary sources to provide all essential amino acids [37]. This study did not assess protein quality, as the NNR 2023 does not provide recommendations for specific amino acids. Seafood is an important source of n-3 fatty acids, so a transition to more plant-based diets may imply an increased risk of lower intake levels. Nevertheless, in this study intake of both total n-3 fatty acids and ALA reached recommended intake across all quintiles of dietary environmental impact. While lower environmental impact was associated with lower intake of total n-3 fatty acids, the difference in intake between the highest and lowest impact groups was small.

Lower levels of certain minerals are raised as a concern for diets with a lower environmental impact, as meat, dairy and seafood are major sources in affluent countries like Sweden [30]. The risk of inadequate levels of iron is of special concern considering that meat, and particularly red meat, is a main dietary source of iron, and a prevalence of low iron intake and status has been observed among Swedish women [30, 32, 38]. In this study, no association was found between dietary environmental impact and intake of iron in women, and the risk of iron deficiency is of less concern due to the older population studied not including fertile women. In fact, several studies have found associations between higher intakes of iron in diets with a lower climate impact [8, 10]. Importantly, these studies, like ours, did not consider the lower bioavailability of minerals in plant-based compared to animal-based foods, attributed by multiple factors, including the form (e.g., heme vs. non-heme iron) and presence of dietary inhibitors (e.g. phytic acid and polyphenols).

### Implications of including a broad environmental perspective

While adopting a broad environmental perspective in dietary sustainability assessments is essential to avoid trade-offs between environmental goals, such approaches remain limited in the scientific literature [20]. Hallström et al. [6] previously evaluated the environmental impact of diets in the same study population, showing that food groups differed in their impact across environmental indicators. Animal-based foods dominated impacts on GHG emissions, cropland use, and N and P application, whereas plant-based and discretionary foods had a greater contribution to consumptive water use and extinction rate [6].

This study assumed equal weighting of six environmental indicators, and individuals in the lowest quintile of the aggregated score (Q1) also ranked lowest across all individual indicators (Fig. S1). The requirement for relatively lower impact across multiple indicators may explain differences from previous assessments focused solely on climate impact. For example, fresh fruit was previously estimated to contribute only 3% of total dietary climate impact but 19% and 27% of total freshwater use and extinction rate, respectively, in the studied population [6].

To assess the impact of using an aggregated environmental score versus individual indicators, a sensitivity analysis was conducted examining associations between selected food groups and dietary environmental impact for freshwater use, extinction rate and GHG emissions. The food groups — vegetables, ‘fruits and berries’, ‘bread, grains, cereals, rice and pasta’ and ‘sweets and snacks’ — were considered based on previous findings showing their relatively higher contributions to freshwater use and extinction rate compared to GHG emissions [6]. Results showed that energy standardised intake of fruits and berries was positively associated with freshwater use and extinction rate but negatively associated with GHG emissions (significant only in women). For the other food groups, associations with dietary environmental impact were consistent with those observed using the aggregated environmental score, regardless of the specific environmental indicator used.

In this study, GHG emissions, cropland use, and P and N application aligned closely with the aggregated score, while freshwater use and extinction rate showed some deviation (Figure S1). Although strong correlation among a broad range of environmental impacts have been observed in Nordic dietary contexts [39], trade-offs — particularly between water use and other indicators are described in the literature [7, 40–43]. Some studies also reported higher ecosystem toxicity [43] and biodiversity damage [42] from plant-rich diets with otherwise lower environmental impact. Furthermore, associations between health and environmental benefits appear strongest for GHG emissions, moderate for cropland, application of P and N, and weakest for freshwater use [41]. These findings underscore the need for broad sustainability analyses to minimise trade-offs in dietary policies.

### Uncertainties and method consideration

This study´s strengths include using self-reported dietary data from large population-based cohorts and a broad environmental perspective based on LCA data representative for Swedish food consumption. The combined health and environmental perspective allowed evaluation of potential co-benefits and trade-offs between sustainability perspectives, and the benchmarking against NNR 2023 provides fresh insights relevant for ongoing efforts in dietary guidelines and sustainable diet policies.

The study also has limitations. The observational data used means results reflect associations, not causal relationships. The study population of Swedish individuals aged 56–70 may not be representative for other age groups as dietary habits and nutrient requirements differ between age groups. For example, younger adults (18–30 years) in Sweden are found to have less healthy diets compared to older generations, with lower consumption of fruits and vegetables, fish and whole grains and higher intake of sweetened beverages and fast food [30]. Additionally, differences in dietary habits and food production systems, may limit generalizability to other regions. Furthermore, the dietary data from 2009 do not capture changes in dietary patterns over the past 15 years. National consumption statistics indicate relatively minor changes (< 10%) in consumption of most foods during this period [6]. Specifically, per capita consumption of charcuterie, milk, cream, and beer decreased, while vegetable consumption increased [44]. Nevertheless, the inability to capture newer trends in consumption and production, including emerging food products on the market and changes in fortification levels, is a major limitation and future studies would benefit from using more updated dietary data. The reliance on self-reported dietary data introduces uncertainty, particularly due to recall-bias and misreporting [45]. A large uncertainty also exists in the estimates of absolute intake amounts from dietary data based on FFQs. To reduce uncertainties linked to overreporting and/or underreporting of dietary intake, assessments of diet quality were made per isocaloric energy intake based on recommended energy intake levels. However, total energy intake strongly influences food and nutrient intake, and many individuals in practice eat more or less energy than recommended. Intake of some food groups, e.g., pulses, was low, which limited the possibility to assess associations with dietary environmental impact. Moreover, vegetable oils and spreads were assessed in the FFQ using only qualitative questions and were then incorporated into estimation of e.g., energy intake and fatty acids, based on standard recipes. As a results, quantitative intake estimates for these items could not be provided. Furthermore, nutrient intake from dietary supplements was not accounted for which may underestimate actual nutrient intake. For instance, supplements have been estimated to contribute approximately 9–19% of total vitamin D intake in a comparable Swedish adult cohort [46].

The study's scope should also be considered, as not all sustainability aspects were addressed. Some nutrients were excluded due to data limitations, and bioavailability and nutritional status were not considered but are important for future research. All environmental aspects were not assessed, and there is inherent uncertainty in the methods and data used, particularly regarding extinction rate and freshwater use [6]. The selection and equal weighting of environmental indicators can have major implications on results. Our results indicate strong correlations among four of six indicators assessed — GHG emission, cropland use, application of P and N — raising questions about the necessity of including all in an aggregated score. Recent guidance on indicator choice for environmental assessments of diets recommends including indicators for climate change, biosphere integrity (e.g. biodiversity impact from land use), water consumption, novel entities (e.g., ecotoxicity or pesticide use), and exploitation of wild fish stocks to capture key environmental trade-offs [47]. Various approaches to apply weighting factors for environmental indicators have been suggested. Weighting factors may for example be based on the current performance in relation to set goals or value-based preferences based on expert opinion or monetary valuation [48]. Previous studies indicated that average Swedish diets exceed *per capita* planetary boundaries for all the environmental indicators assessed in this study, except for freshwater use, with N and P application, extinction rate, GHG emissions exceeding the boundaries by over three-fold [5, 6, 49]. An aggregated environmental score based the current performance in relation to goal could therefore reduce the relative impact of freshwater and cropland use. Although the EU-PEF framework, aiming to standardize LCA methodology, proposes weighting values for composite environmental scores [50], no standardized method is recommended for dietary studies. Further research is needed to clarify how indicator selection and weighting influence dietary sustainability assessments and inform policy.

## Conclusions

Diet change can be a powerful tool for achieving environmental and health goals. This study indicates that self-selected diets with a lower impact on multiple environmental indicators may be both positively and negatively associated with diet quality. Diets with lower environmental impact were characterized by *lower* intakes of red meat, processed meat, sugar-sweetened beverages, high-fat dairy products, saturated fat, sodium and alcohol, and *higher* intake of whole grains, fibre and polyunsaturated fat which align with dietary guidelines and have great potential for improving health. To minimize negative health effects from diets with lower environmental impact, it is crucial to limit intake of foods with low nutritional value (e.g., sweets and snacks), while promoting the intake of healthy plant-based foods (e.g., vegetables, fruits and berries, legumes, nuts and seeds) to recommended levels. Potential risks associated with a lower intake of some micronutrients should be considered in policies for sustainable diets, especially among population groups with special requirements (e.g., children, adolescents, fertile and pregnant women, the elderly). Impact of nutrient status and bioavailability are other aspects to consider in future studies to better understand the nutritional and health implications of sustainable diets with low environmental impact.

## Supplementary Information

Below is the link to the electronic supplementary material.

Supplementary file 1.
