# Supplementary material for: Co-benefits and trade-offs between environmental impact and diet quality: insights from observational dietary data in a Swedish population
Source: Eur J Nutr. 2026 Jan 16;65(1):31. doi: 10.1007/s00394-025-03855-y (PMC12811323; doi:10.1007/s00394-025-03855-y)
Supplement: Supplementary file 1 [file 394_2025_3855_MOESM1_ESM.docx]

**Supplementary materials**

# **Co-benefits and trade-offs between environmental impact and diet quality: Insights from observational dietary data in a Swedish population**

# European Journal of Nutrition

Elinor Hallström*^a^, Niclas Håkansson^b^, Ulf Sonesson^a^, Agneta Åkesson^b,c^, Alicja Wolk^b^

^a^ Department of Food and Agriculture, Research Institutes of Sweden (RISE), Lund/Göteborg, Sweden.

**^b^**Institute of Environmental Medicine, Karolinska Institute, Stockholm, Sweden.

**^c^** Department of Surgical Sciences, Uppsala University, Uppsala, Sweden.

*Corresponding author: [Elinor.Hallstrom@ri.se](mailto:Elinor.Hallstrom@ri.se)

#

1. **Method description of environmental score**

A detailed step-by-step description of the method used to calculate the diet's aggregate environmental impact is provided below:

1. The mean dietary environmental impact per 1000 kcal of all study participants was quantified for each of the six environmental indicators separately.
2. Population-based quintiles, for women and men, were constructed for each environmental indicator based on the results from step 1
3. The dietary environmental impact of all study participants was assessed against cut-off values based on the population-based quintiles from step 2, for each environmental indicator separately.
4. For each environmental indicator, a value of 1-5 was assigned based on the cut-off values from step 3, reflecting the individuals relative impact compared to the total study population. As illustrated below, one point was assigned to study participants in the first quintile (with lowest impact), two points to participants in the second quintile, and so on.

*Points assigned for individual environmental indicators*

Q1 – 1 point

Q2 – 2 points

Q3 – 3 points

Q4 – 4 points

Q5 – 5 points

1. The points for each of environmental indicator, described in step 4, were added resulting in an aggregated score for each participant ranging from 6 (lowest impact, i.e., 1 point assigned for each of the six indicators) to 30 (highest impact, i.e., 5 points assigned for each of the six indicators), see Table S1.
2. Population-based near quintiles, for women and men, were constructed based on the aggregated environmental scores from step 5, where Q1 and Q5 represent the mean score of study participants with the lowest and highest aggregated environmental impact from the diet, respectively.

**Table S1. Distribution of men and women in quintiles of dietary environmental impact**

|  |  |  | | **Men** | | |  | | **Women** | | |
| --- | --- | --- | --- | --- | --- | --- | --- | --- | --- | --- | --- |
| **Quintile** | **Environmental score** | **Frequency** | **Percent** | | **Q%** | **Age**  **Mean (SD)** | **Frequency** | **Percent** | | **Q%** | **Age**  **Mean (SD)** |
| Q1 | 6 | 455 | 3.0 | | 19.2 | 63.9 (4.10) | 287 | 2.0 | | 16.8 | 65.5 (2.92) |
|  | 7 | 460 | 3.0 | |  |  | 285 | 2.0 | |  |  |
|  | 8 | 418 | 2.7 | |  |  | 372 | 2.6 | |  |  |
|  | 9 | 489 | 3.2 | |  |  | 387 | 2.7 | |  |  |
|  | 10 | 562 | 3.7 | |  |  | 533 | 3.7 | |  |  |
|  | 11 | 563 | 3.7 | |  |  | 548 | 3.8 | |  |  |
| Q2 | 12 | 663 | 4.3 | | 18.8 | 63.7 (3.99) | 636 | 4.4 | | 20.1 | 65.2 (2.93) |
|  | 13 | 704 | 4.6 | |  |  | 717 | 5.0 | |  |  |
|  | 14 | 717 | 4.7 | |  |  | 755 | 5.3 | |  |  |
|  | 15 | 789 | 5.2 | |  |  | 780 | 5.4 | |  |  |
| Q3 | 16 | 724 | 4.7 | | 24.4 | 63.6 (3.98) | 751 | 5.2 | | 26.7 | 65.0 (2.89) |
|  | 17 | 761 | 5.0 | |  |  | 795 | 5.5 | |  |  |
|  | 18 | 745 | 4.9 | |  |  | 788 | 5.5 | |  |  |
|  | 19 | 755 | 4.9 | |  |  | 758 | 5.3 | |  |  |
|  | 20 | 747 | 4.9 | |  |  | 738 | 5.1 | |  |  |
| Q4 | 21 | 730 | 4.8 | | 18.1 | 63.4 (3.86) | 732 | 5.1 | | 19.1 | 64.6 (2.86) |
|  | 22 | 677 | 4.4 | |  |  | 680 | 4.7 | |  |  |
|  | 23 | 667 | 4.4 | |  |  | 669 | 4.7 | |  |  |
|  | 24 | 700 | 4.6 | |  |  | 667 | 4.6 | |  |  |
| Q5 | 25 | 571 | 3.7 | | 19.5 | 63.1 (3.86) | 584 | 4.1 | | 17.1 | 64.4 (2.78) |
|  | 26 | 524 | 3.4 | |  |  | 522 | 3.6 | |  |  |
|  | 27 | 530 | 3.5 | |  |  | 435 | 3.0 | |  |  |
|  | 28 | 496 | 3.2 | |  |  | 358 | 2.5 | |  |  |
|  | 29 | 455 | 3.0 | |  |  | 337 | 2.4 | |  |  |
|  | 30 | 406 | 2.7 | |  |  | 248 | 1.7 | |  |  |

**Table S2. Categorisation of food groups assessed**

| **Food group categorization** | **Specification of included food items** |
| --- | --- |
| **RED MEAT** |  |
| Beef | Beef/veal |
| Pork | Pork |
| Lamb, game, offal and unspecified red meat | Lamb, moose, deer, liver/kidney |
| Processed red meat | Sausage, cold cuts meat (e.g. ham/turkey), cold cuts sausage (e.g. salami), bacon, liver paté, blood pudding/sausage. |
| **WHITE MEAT** |  |
| White meat | Chicken, other poultry |
| **EGGS** |  |
| Eggs | Eggs |
| **DAIRY PRODUCTS** |  |
| Milk | Milk |
| Yoghurt | Yoghurt, sour milk, yoghurt for cooking |
| Cheese | Cottage cheese/quark, cream cheese, hard cheese, dessert cheese |
| Cream and crème fraiche | Creme fraiche, double creme, single cream, sour cream |
| **SEAFOOD** |  |
| Fatty fish | Herring, mackerel, salmon, trout, sardines |
| Other seafood | Cod, saithe, Alaska pollock, plaice, tuna, pike, perch, fish sticks, caviar, shellfish, other fish |
| **BREAD, GRAINS & CERALS, RICE, PASTA** |  |
| Bread | White bread, fiber enriched bread, whole grain bread, crisp bread |
| Grains, porridge and cereals | Oatmeal/rye porridge, other porridge/gruel, muesli, breakfast cereal, wheat or oat bran |
| Pasta, rice and couscous | Spaghetti, macaroni, pasta, couscous, bulgur, rice |
| **VEGETABLES** |  |
| Roots and tubers (excl. potatoes) | Carrot, beetroot |
| Salad vegetables | Lettuce, tomato/tomato juice, peppers, spinach |
| Onions | Onion, leek, garlic, chive |
| Cabbages | Cabbage, cauliflower, broccoli, brussels sprouts |
| Fresh legumes | Green peas, haricots verts |
| Other vegetables and herbs | Mixed frozen vegetables, avocado, corn, olives, other vegetables |
| **POTATOES** |  |
| Potatoes | Boiled potatoes, fried potatoes, baked/mashed potatoes, French fries |
| **PULSES/LEGUMES** |  |
| Pulses | Beans, lentils, chickpeas |
| **FRUITS AND BERRIES** |  |
| Fresh fruits | Orange, other citrus fruit, apple, pears, banana, other fruit |
| Berries | Berries (fresh or frozen) |
| Dried fruits | Prunes, raisins, dried apricots, other dried fruits |
| **NUTS AND SEEDS** |  |
| Nuts | Peanuts, almonds, other nuts |
| Seeds | Flax seeds, sesame seeds, sunflower seeds, pumpkin seeds, other seeds |
| **SWEETS AND SNACKS** |  |
| Sugar and honey | Sugar, honey |
| Jam and fruit soups | Jam, fruit soup |
| Cookies, cakes and pastries | Buns, cookies, biscuits, wafers, cakes, pastries |
| Chocolate and candy | Chocolate, candy |
| Ice cream | Ice cream, sorbet |
| Salty snacks | Chips, popcorn, cheese puffs |
| **NON-ALCOHOLIC DRINKS** |  |
| Juice | Orange juice, grapefruit juice, other fruit juice |
| Soda and squash | Soda, squash |
| Tea and coffee | Tea, coffee |
| **ALCOHOLIC DRINKS** |  |
| Beer | Beer |
| Wine | Wine |
| Strong wine | Liqueur, sherry, port wine |
| Spirits | Spirits |
| **OTHER FOODS** |  |
| Other foods | Salad dressing, mayonnaise, ketchup, dried herbs, cinnamon, pepper, table salt |

**Table S3. Quintile cut-off values for environmental impact in women and men**

|  | **Q1** | **Q2** | **Q3** | **Q4** | **Q5** |
| --- | --- | --- | --- | --- | --- |
| **Women** | | | | | |
| **GHGE**  (kg CO_2_e per 1000 kcal) | <2.38 | 2.38.-2.60 | 2.60-2.81 | 2.81-3.08 | >3.08 |
| **Cropland use**  (m^2^ per 1000 kcal) | <2.84 | 2.84-3.12 | 3.12-3.38 | 3.38-3.75 | >3.75 |
| **N application**  (kg N per 1000 kcal) | <0.052 | 0.052-0.059 | 0.059-0.065 | 0.065-0.074 | >0.074 |
| **P application**  (kg P per 1000 kcal) | <0.0037 | 0.0037-0.0040 | 0.0040-0.0044 | 0.0044-0.0048 | >0.0048 |
| **Consumptive water use**  (m^3^ per 1000 kcal) | <0.051 | 0.051-0.061 | 0.061-0.072 | 0.072-0.084 | >0.084 |
| **Extinction rate**  (E/MSY per 1000 kcal) | <5.89 E^-12^ | 5.89 E^-12^  -7.18-E^-12^ | 7.18 E^-12^  -8.49. E^-12^ | 8.49 E^-12^  -1.03 E^-11^ | >1.03 E^-11^ |
| **Men** | | | | | |
| **GHGE**  (kg CO_2_e per 1000 kcal) | <2.36 | 2.36-2.57 | 2.57-2.77 | 2.77-3.04 | >3.04 |
| **Cropland use**  (m^2^ per 1000 kcal) | <2.82 | 2.82-3.11 | 3.11-3.37 | 3.37-3.70 | >3.70 |
| **N application**  (kg N per 1000 kcal) | <0.053 | 0.053-0.060 | 0.060-0.066 | 0.066-0.074 | >0.074 |
| **P application**  (kg P per 1000 kcal) | <0.0035 | 0.0035-0.0039 | 0.0039-0.0042 | 0.0042-0.0047 | >0.0047 |
| **Consumptive water use**  (m^3^ per 1000 kcal) | <0.041 | 0.041-0.049 | 0.049-0.057 | 0.057-0.068 | >0.068 |
| **Extinction rate**  (E/MSY per 1000 kcal) | <4.71 E^-12^ | 4.71 E^-12^  -5.83 E^-12^ | 5.83 E^-12^  -6.98 E^-12^ | 6.98 E^-12^  -8.68 E^-12^ | >8.68 E^-12^ |

**Table S4. Mean food group intake, g per 1000 kcal (SD) in women, by quintiles of dietary environmental impact and p-values for linear trend. Q5 represents diets with highest environmental impact.**

|  | **Quintiles of dietary environmental impact** | | | | | **Linear regression** |
| --- | --- | --- | --- | --- | --- | --- |
|  | **Q1** | **Q2** | **Q3** | **Q4** | **Q5** | **p-value** |
| **Women (n)** | 2412 | 2888 | 3830 | 2748 | 2484 |  |
| ***Red meat*** | 28 (14) | 31 (15) | 35 (16) | 40 (18) | 43 (19) | <.0001 |
| Beef | 4.6 (2.8) | 5.4 (3.0) | 6.7 (3.3) | 7.9 (4.4) | 9.3 (5.7) | <.0001 |
| Pork | 3.1 (1.9) | 3.4 (1.9) | 3.9 (2.1) | 4.3 (2.3) | 4.7 (2.9) | <.0001 |
| Lamb, game, offals and  unprocessed red meat | 1.8 (2.4) | 2.3 (3.9) | 2.7 (3.0) | 3.0 (3.5) | 4.0 (4.5) | <.0001 |
| Processed red meat | 19 (12) | 20 (12) | 22 (14) | 24 (15) | 25 (15) | <.0001 |
| ***White meat*** | 6.4 (5.4) | 7.5 (5.6) | 8.6 (6.0) | 9.8 (6.5) | 11 (7.4) | <.0001 |
| ***Eggs*** | 5.9 (4.7) | 6.5 (4.8) | 6.9 (5.3) | 7.5 (5.9) | 8.2 (7.0) | <.0001 |
| ***Dairy products*** | 199 (113) | 212 (118) | 232 (141) | 246 (142) | 249 (131) | <.0001 |
| Milk | 73 (69) | 76 (70) | 81 (86) | 81 (86) | 73 (85) | 0.3355 |
| Yoghurt | 96 (96) | 101 (99) | 111 (115) | 108 (112) | 102 (102) | 0.0035 |
| Cheese | 26 (16) | 30 (19) | 35 (23) | 51 (48) | 68 (65) | <.0001 |
| Cream and crème fraiche | 4.9 (3.4) | 5.3 (3.5) | 5.7 (3.7) | 6.1 (4.2) | 6.6 (4.4) | <.0001 |
| ***Seafood*** | 25 (15) | 27 (15) | 28 (14) | 29 (15) | 31 (16) | <.0001 |
| Fatty fish | 11 (8.8) | 11 (8.3) | 12 (8.3) | 12 (8.6) | 13 (8.6) | <.0001 |
| Other seafood | 14 (10) | 15 (10) | 16 (9.4) | 17 (10) | 18 (11) | <.0001 |
| ***Bread, grains, cereals, rice and pasta*** | 136 (51) | 119 (43) | 111 (41) | 102 (41) | 92 (42) | <.0001 |
| Bread | 69 (32) | 55 (23) | 50 (21) | 45 (20) | 39 (19) | <.0001 |
| Grain, porridge and cereals | 42 (41) | 39 (38) | 36 (36) | 31 (34) | 28 (34) | <.0001 |
| Pasta, rice and couscous | 26 (21) | 25 (16) | 26 (17) | 26 (17) | 25 (17) | 0.7381 |
| ***Vegetables*** | 106 (59) | 122 (62) | 127 (65) | 135 (66) | 150 (71) | <.0001 |
| Roots and tubers (excl. potatoes) | 15 (12) | 17 (13) | 17 (13) | 17 (13) | 18 (14) | <.0001 |
| Salad vegetables | 36 (27) | 42 (29) | 45 (31) | 48 (33) | 53 (37) | <.0001 |
| Onions | 9.6 (7.0) | 11 (7.1) | 12 (7.7) | 13 (7.9) | 14 (8.8) | <.0001 |
| Cabbage | 18 (18) | 22 (20) | 23 (22) | 25 (23) | 30 (27) | <.0001 |
| Fresh legumes | 5.0 (5.8) | 5.7 (6.2) | 5.6 (6.0) | 6.0 (6.0) | 6.2 (6.0) | <.0001 |
| Other vegetables and herbs | 22 (19) | 24 (18) | 25 (19) | 26 (19) | 29 (19) | <.0001 |
| ***Potatoes*** | 47 (32) | 46 (28) | 46 (26) | 46 (27) | 42 (27) | <.0001 |
| ***Pulses*** | 5.9 (6.7) | 6.1 (6.1) | 6.0 (5.7) | 6.0 (5.7) | 5.8 (5.5) | 0.5412 |
| ***Fruits and berries*** | 81 (47) | 108 (64) | 110 (67) | 111 (71) | 124 (70) | <.0001 |
| Fresh fruits | 68 (42) | 92 (57) | 95 (61) | 95 (66) | 107 (65) | <.0001 |
| Berries | 9.8 (13) | 12 (17) | 12 (16) | 13 (17) | 14 (19) | <.0001 |
| Dried fruit | 3.3 (6.1) | 4.0 (6.7) | 3.2 (5.4) | 2.6 (4.6) | 2.5 (4.7) | <.0001 |
| ***Nuts and seeds*** | 3.1 (4.2) | 4.8 (6.1) | 4.4 (5.3) | 4.1 (5.1) | 4.9 (5.7) | <.0001 |
| Nuts | 1.7 (2.6) | 2.7 (4.1) | 2.4 (3.2) | 2.2 (3.1) | 2.7 (3.5) | <.0001 |
| Seeds | 1.4 (2.8) | 2.1 (3.6) | 2.0 (3.5) | 1.8 (3.4) | 2.2 (3.7) | <.0001 |
| ***Sweets and snacks*** | 30 (21) | 29 (19) | 27 (18) | 25 (18) | 21 (15) | <.0001 |
| Sugar and honey | 3.4 (6.2) | 2.4 (5.0) | 1.8 (4.4) | 1.5 (3.6) | 1.1 (3.7) | <.0001 |
| Jam and fruit soups | 11 (15) | 10 (14) | 10 (14) | 8.8 (13) | 6.9 (11) | <.0001 |
| Cookies, cakes and pastries | 9.3 (8.5) | 8.7 (7.3) | 7.8 (6.6) | 7.0 (6.1) | 6.0 (5.2) | <.0001 |
| Chocolate and candy | 2.7 (2.8) | 3.0 (3.1) | 3.4 (3.6) | 3.4 (3.7) | 3.2 (3.4) | <.0001 |
| Ice cream | 3.6 (4.3) | 3.8 (4.6) | 3.8 (4.5) | 3.9 (4.5) | 3.7 (4.4) | 0.4101 |
| Salty snacks | 0.3 (0.8) | 0.3 (0.7) | 0.4 (0.8) | 0.4 (0.9) | 0.3 (0.8) | 0.0078 |
| ***Non-alcoholic drinks (excl. water)*** | 350 (200) | 367 (174) | 391 (186) | 424 (207) | 531 (300) | <.0001 |
| Juice | 12 (21) | 16 (27) | 18 (29) | 18 (31) | 19 (33) | <.0001 |
| Soda and squash | 23 (77) | 21 (57) | 26 (81) | 30 (90) | 49 (148) | <.0001 |
| Tea and coffee | 315 (187) | 330 (169) | 347 (169) | 376 (190) | 463 (269) | <.0001 |
| ***Alcoholic drinks*** | 27 (31) | 34 (36) | 39 (39) | 41 (41) | 45 (43) | <.0001 |
| Beer | 11 (19) | 11 (19) | 12 (21) | 11 (20) | 9.1 (17) | 0.0012 |
| Wine | 15 (21) | 22 (27) | 26 (30) | 30 (33) | 35 (37) | <.0001 |
| Strong wine | 0.3 (0.9) | 0.3 (0.9) | 0.4 (1.0) | 0.4 (1.0) | 0.3 (0.9) | 0.3839 |
| Spirits | 0.4 (1.4) | 0.5 (1.5) | 0.6 (1.4) | 0.6 (1.5) | 0.6 (1.6) | <.0001 |
| ***Other foods*** | 2.6 (2.3) | 2.9 (2.1) | 3.1 (2.4) | 3.2 (2.4) | 3.5 (2.9) | <.0001 |

**Table S5. Mean food group intake, g per 1000 kcal (SD) in men, by quintiles of dietary environmental impact and p-values for linear trend. Q5 represents diets with highest environmental impact.**

|  | **Quintiles of dietary environmental impact** | | | | | **Linear regression** |
| --- | --- | --- | --- | --- | --- | --- |
|  | **Q1** | **Q2** | **Q3** | **Q4** | **Q5** | **p-value** |
| **Men (n)** | 2947 | 2873 | 3732 | 2774 | 2982 |  |
| ***Red meat*** | 30 (13) | 34 (14) | 40 (14) | 45 (16) | 55 (21) | <.0001 |
| Beef | 5.3 (3.0) | 6.6 (3.4) | 8.0 (3.8) | 9.6 (4.8) | 13 (7.3) | <.0001 |
| Pork | 4.2 (2.7) | 4.9 (2.8) | 5.5 (2.8) | 6.3 (3.3) | 7.6 (4.7) | <.0001 |
| Lamb, moose, offals and  unprocessed red meat | 2.2 (3.0) | 3.1 (3.6) | 3.9 (4.1) | 4.8 (4.7) | 7.6 (7.9) | <.0001 |
| Processed red meat | 18 (11) | 20 (11) | 22 (12) | 24 (14) | 27 (16) | <.0001 |
| ***White meat*** | 4.8 (4.2) | 5.9 (4.7) | 6.4 (5.0) | 7.2 (5.1) | 8.7 (6.5) | <.0001 |
| ***Eggs*** | 6.3 (5.5) | 7.1 (5.7) | 7.7 (6.4) | 8.2 (7.0) | 9.5 (8.5) | <.0001 |
| ***Dairy products*** | 197 (119) | 211 (132) | 228 (146) | 234 (149) | 224 (138) | <.0001 |
| Milk | 92 (89) | 98 (98) | 107 (111) | 112 (115) | 104 (113) | <.0001 |
| Yoghurt | 77 (90) | 82 (100) | 87 (105) | 83 (99) | 78 (93) | 0.6113 |
| Cheese | 24 (15) | 27 (17) | 30 (19) | 34 (27) | 36 (31) | <.0001 |
| Cream and crème fraiche | 3.9 (3.1) | 4.6 (3.4) | 4.8 (3.3) | 5.3 (3.7) | 6.0 (4.3) | <.0001 |
| ***Seafood*** | 20 (12) | 22 (12) | 24 (13) | 26 (14) | 27 (14) | <.0001 |
| Fatty fish | 8.3 (6.9) | 9.3 (7.1) | 9.8 (7.2) | 10 (7.6) | 11 (8.1) | <.0001 |
| Other seafood | 12 (7.5) | 13 (7.7) | 14 (8.3) | 15 (9.6) | 16 (9.9) | <.0001 |
| ***Bread, grains, cereals, rice and pasta*** | 144 (45) | 124 (39) | 115 (36) | 108 (35) | 97 (37) | <.0001 |
| Bread | 99 (39) | 79 (29) | 71 (27) | 64 (26) | 54 (25) | <.0001 |
| Grain, porridge and cereals | 22 (28) | 21 (24) | 19 (23) | 18 (22) | 17 (23) | <.0001 |
| Pasta, rice and couscous | 22 (19) | 24 (21) | 25 (17) | 26 (18) | 26 (19) | <.0001 |
| ***Vegetables*** | 63 (38) | 76 (45) | 78 (42) | 81 (46) | 94 (52) | <.0001 |
| Roots and tubers (excl. potatoes) | 9.6 (8.8) | 11 (9.2) | 11 (8.7) | 11 (8.6) | 11 (9.1) | <.0001 |
| Salad vegetables | 20 (16) | 25 (18) | 26 (19) | 27 (19) | 31 (23) | <.0001 |
| Onions | 6.8 (4.8) | 8.3 (5.5) | 8.8 (5.7) | 9.5 (5.7) | 12 (7.1) | <.0001 |
| Cabbage | 12 (12) | 14 (17) | 14 (14) | 16 (16) | 19 (20) | <.0001 |
| Fresh legumes | 4.0 (5.2) | 4.6 (5.2) | 4.7 (5.1) | 4.7 (5.6) | 5.1 (5.6) | <.0001 |
| Other vegetables and herbs | 11 (10) | 13 (11) | 13 (11) | 14 (11) | 16 (12) | <.0001 |
| ***Potatoes*** | 51 (30) | 52 (29) | 53 (28) | 54 (29) | 55 (32) | <.0001 |
| ***Pulses*** | 6.0 (6.1) | 6.6 (6.8) | 6.8 (6.4) | 6.4 (6.0) | 6.9 (6.2) | <.0001 |
| ***Fruits and berries*** | 45 (31) | 59 (42) | 60 (45) | 61 (46) | 65 (46) | <.0001 |
| Fresh fruits | 38 (28) | 50 (38) | 52 (41) | 52 (42) | 57 (43) | <.0001 |
| Berries | 5.1 (8.0) | 6.3 (9.2) | 6.4 (9.0) | 6.5 (9.4) | 6.7 (9.7) | <.0001 |
| Dried fruit | 1.8 (3.5) | 2.4 (4.5) | 2.0 (4.0) | 1.9 (4.1) | 1.6 (3.0) | <.0001 |
| ***Nuts and seeds*** | 1.1 (2.0) | 1.6 (3.1) | 1.5 (2.7) | 1.5 (2.6) | 1.6 (2.9) | <.0001 |
| Nuts | 0.5 (0.9) | 0.6 (1.2) | 0.6 (1.1) | 0.6 (1.0) | 0.7 (1.2) | <.0001 |
| Seeds | 0.6 (1.7) | 1.0 (2.6) | 0.8 (2.3) | 0.9 (2.2) | 0.9 (2.5) | 0.0002 |
| ***Sweets and snacks*** | 30 (21) | 31 (19) | 30 (19) | 27 (17) | 26 (18) | <.0001 |
| Sugar and honey | 3.6 (5.6) | 3.2 (5.4) | 2.7 (5.0) | 2.2 (4.4) | 1.8 (4.2) | <.0001 |
| Jam and fruit soups | 9.9 (14) | 10 (13) | 10 (14) | 9.1 (12) | 8.9 (14) | 0.0001 |
| Cookies, cakes and pastries | 10 (10) | 10 (8.9) | 9.3 (8.2) | 8.2 (7.5) | 7.1 (6.9) | <.0001 |
| Chocolate and candy | 2.3 (2.4) | 2.7 (3.0) | 2.8 (3.1) | 2.9 (2.9) | 2.9 (3.1) | <.0001 |
| Ice cream | 3.7 (4.4) | 4.0 (4.2) | 4.2 (4.5) | 4.3 (4.6) | 4.2 (4.7) | <.0001 |
| Salty snacks | 0.5 (1.2) | 0.7 (1.5) | 0.7 (1.2) | 0.7 (1.4) | 0.7 (1.5) | <.0001 |
| ***Non-alcoholic drinks (excl. water)*** | 309 (160) | 347 (172) | 372 (189) | 410 (204) | 525 (293) | <.0001 |
| Juice | 12 (22) | 17 (26) | 20 (29) | 20 (30) | 23 (34) | <.0001 |
| Soda and squash | 33 (77) | 41 (93) | 51 (109) | 62 (134) | 96 (201) | <.0001 |
| Tea and coffee | 263 (146) | 288 (152) | 302 (159) | 328 (172) | 407 (240) | <.0001 |
| ***Alcoholic drinks*** | 52 (52) | 59 (52) | 64 (55) | 69 (60) | 73 (61) | <.0001 |
| Beer | 38 (45) | 39 (42) | 41 (44) | 42 (45) | 40 (45) | 0.0041 |
| Wine | 12 (18) | 18 (23) | 20 (25) | 25 (31) | 30 (34) | <.0001 |
| Strong wine | 0.3 (0.9) | 0.4 (1.2) | 0.4 (1.1) | 0.4 (1.1) | 0.4 (1.0) | 0.0007 |
| Spirits | 1.8 (3.3) | 2.1 (3.4) | 2.4 (3.7) | 2.6 (3.9) | 2.9 (4.1) | <.0001 |
| ***Other foods*** | 3.2 (2.7) | 3.5 (2.9) | 3.8 (2.9) | 4.1 (3.0) | 4.5 (3.7) | <.0001 |

**Table S6. Nutrient intake per 1000 kcal in women, mean intake (SD), by quintiles of dietary environmental impact and p-values for linear regression. Q5 represent diets with highest environmental impact.**

|  | **Quintiles of dietary environmental impact** | | | | | **Linear regression** |
| --- | --- | --- | --- | --- | --- | --- |
|  | **Q1** | **Q2** | **Q3** | **Q4** | **Q5** | **p** |
| **Women (n)** | 2412 | 2888 | 3830 | 2748 | 2482 |  |
| ***Macronutrients*** | | | | | | |
| Protein, g | 41.4 (4.6) | 42.9 (4.9) | 45.4 (5.3) | 48.4 (6.7) | 51.2 (7.2) | <0.0001 |
| Carbohydrates, g | 120 (13) | 114 (14) | 110 (14) | 106 (16) | 101 (19) | <0.0001 |
| Dietary fibre, g | 15.0 (3.3) | 14.7 (3.2) | 14.1 (3.1) | 13.5 (3.2) | 13.3 (3.1) | <0.0001 |
| Whole grains, g | 50.6 (20) | 44.3 (16) | 41.1 (15) | 37.6 (16) | 34.2 (15) | <0.0001 |
| Sucrose, g | 15.9 (8.7) | 15.9 (6.8) | 15.4 (6.7) | 14.6 (6.5) | 14.4 (6.9) | <0.0001 |
| Total fat, g | 34.2 (5.6) | 35.4 (5.7) | 35.9 (5.7) | 36.7 (6.3) | 37.0 (7.2) | <0.0001 |
| SFA^1^, g | 14.0 (3.3) | 14.4 (3.5) | 14.9 (3.5) | 15.4 (3.8) | 15.4 (3.9) | <0.0001 |
| MUFA^2^, g | 11.3 (2.1) | 11.9 (2.2) | 12.0 (2.1) | 12.3 (2.3) | 12.5 (2.6) | <0.0001 |
| PUFA^3^, g | 5.80 (1.5) | 5.98 (1.7) | 5.86 (1.6) | 5.72 (1.5) | 5.83 (1.8) | 0.0275 |
| LA^4^, g | 4.23 (1.1) | 4.36 (1.2) | 4.25 (1.1) | 4.12 (1.1) | 4.18 (1.3) | <0.0001 |
| **ALA^5^, g** | 1.03 (0.5) | 1.06 (0.5) | 1.03 (0.5) | 0.99 (0.5) | 1.02 (0.6) | 0.0030 |
| EPA^6^, g | 0.084 (0.06) | 0.090 (0.06) | 0.093 (0.06) | 0.095 (0.06) | 0.101 (0.06) | <0.0001 |
| DPA^7^, g | 0.029 (0.02) | 0.030 (0.02) | 0.032 (0.02) | 0.034 (0.02) | 0.036 (0.02) | <0.0001 |
| DHA^8^, g | 0.192 (0.13) | 0.205 (0.12) | 0.216 (0.12) | 0.224 (0.12) | 0.239 (0.13) | <0.0001 |
| Tot n-3 fatty acids^9^, g | 1.33 (0.51) | 1.39 (0.56) | 1.37 (0.56) | 1.34 (0.57) | 1.39 (0.62) | 0.0396 |
| Alcohol | 1.98 (2.3) | 2.71 (3.0) | 3.16 (3.3) | 3.49 (3.6) | 3.98 (3.9) | <0.0001 |
| ***Vitamins*** | | | | | | |
| Vitamin A, RE | 640 (401) | 657 (466) | 690 (443) | 725 (496) | 716 (481) | <0.0001 |
| Vitamin D, µg | 3.73 (1.5) | 3.83 (1.3) | 4.03 (1.4) | 4.13 (1.5) | 4.18 (1.5) | <0.0001 |
| Vitamin E, mg | 4.97 (1.1) | 5.32 (1.2) | 5.27 (1.2) | 5.24 (1.2) | 5.45 (1.2) | <0.0001 |
| Thiamin, mg | 0.68 (0.1) | 0.69 (0.1) | 0.70 (0.1) | 0.70 (0.1) | 0.71 (0.1) | <0.0001 |
| Riboflavin, mg | 0.95 (0.2) | 0.97 (0.2) | 1.01 (0.2) | 1.04 (0.2) | 1.05 (0.2) | <0.0001 |
| Vitamin C, mg | 51.5 (22) | 63.0 (26) | 66.4 (28) | 69.2 (29) | 78.4 (30) | <0.0001 |
| Niacin, NE | 18.1 (2.8) | 18.4 (2.5) | 19.1 (2.4) | 20.0 (2.5) | 21.0 (2.6) | <0.0001 |
| Vitamin B6, mg | 1.08 (0.2) | 1.15 (0.2) | 1.16 (0.2) | 1.17 (0.2) | 1.21 (0.2) | <0.0001 |
| Folate, µg | 175 (38) | 185 (47) | 188 (42) | 192 (44) | 202 (48) | <0.0001 |
| Vitamin B12, µg | 3.51 (1.7) | 3.73 (2.2) | 4.02 (1.8) | 4.29 (1.9) | 4.45 (1.9) | <0.0001 |
| ***Minerals*** | | | | | | |
| Iron, mg | 6.59 (1.4) | 6.62 (1.3) | 6.62 (1.3) | 6.59 (1.4) | 6.62 (1.4) | 0.8761 |
| Zinc, mg | 6.02 (0.7) | 6.10 (0.7) | 6.31 (0.7) | 6.51 (0.8) | 6.65 (0.8) | <0.0001 |
| Calcium, mg | 555 (147) | 594 (155) | 638 (183) | 671 (200) | 675 (187) | <0.0001 |
| Phosphorus, mg | 842 (105) | 850 (108) | 876 (125) | 902 (137) | 916 (129) | <0.0001 |
| Potassium, mg | 2529 (1145) | 2406 (902) | 2324 (734) | 2253 (636) | 2280 (562) | <0.0001 |
| Magnesium, mg | 241 (61) | 234 (51) | 228 (43) | 223 (39) | 226 (37) | <0.0001 |
| Selenium, µg | 21.1 (5.4) | 22.6 (5.3) | 24.3 (5.2) | 26.0 (5.6) | 27.5 (6.0) | <0.0001 |
| Sodium, mg | 1404 (206) | 1403 (205) | 1452 (207) | 1523 (219) | 1578 (232) | <0.0001 |
| ^1^SFA ^2^MUFA=Monounsaturated fatty acids. ^3^PUFA=Polyunsaturated fatty acids. ^4^LA= Linoleic acid. ^5^ALA=**α-linolenic acid. ^6^EPA=**Eicosapentaenoic acid. ^7^DPA=Docosapentaenoic acid. ^8^DHA=Docosahexaenoic acid. ^9^Total intake of ALA, EPA, DPA and DHA. | | | | | | |

**Table S7. Nutrient intake per 1000 kcal in men, mean intake (SD), by quintiles of dietary environmental impact and p-values for linear regression. Q5 represent diets with highest environmental impact.**

|  | **Quintiles of dietary environmental impact** | | | | | **Linear regression** |
| --- | --- | --- | --- | --- | --- | --- |
|  | **Q1** | **Q2** | **Q3** | **Q4** | **Q5** | **p** |
| **Men (n)** | 2947 | 2873 | 3732 | 2774 | 2982 |  |
| ***Macronutrients*** | | | | | | |
| Protein, g | 40.7 (4.4) | 42.4 (4.6) | 44.3 (5.1) | 46.4 (5.9) | 48.7 (6.7) | <0.0001 |
| Carbohydrates, g | 122 (13) | 117 (13) | 113 (13) | 109 (15) | 105 (18) | <0.0001 |
| Dietary fibre, g | 14.5 (3.2) | 13.5 (3.2) | 12.8 (3.0) | 12.2 (2.9) | 11.6 (2.9) | <0.0001 |
| Whole grains, g | 53.3 (21) | 46.3 (18) | 42.9 (17) | 39.8 (17) | 35.6 (17) | <0.0001 |
| Sucrose, g | 14.8 (8.4) | 15.6 (8.1) | 15.7 (8.5) | 15.0 (8.5) | 14.8 (9.3) | 0.2800 |
| Total fat, g | 32.7 (5.4) | 33.8 (5.3) | 34.5 (5.4) | 35.1 (5.7) | 35.7 (6.4) | <0.0001 |
| SFA^1^, g | 13.6 (3.3) | 14.2 (3.4) | 14.7 (3.4) | 15.0 (3.5) | 15.0 (3.6) | <0.0001 |
| MUFA^2^, g | 10.6 (1.9) | 11.1 (1.8) | 11.4 (1.8) | 11.7 (2.0) | 12.2 (2.3) | <0.0001 |
| PUFA^3^, g | 5.36 (1.3) | 5.33 (1.3) | 5.26 (1.3) | 5.25 (1.3) | 5.30 (1.4) | 0.0086 |
| LA^4^, g | 3.98 (1.0) | 3.91 (1.0) | 3.84 (1.0) | 3.81 (0.9) | 3.81 (1.0) | <0.0001 |
| **ALA^5^, g** | 0.92 (0.34) | 0.91 (0.38) | 0.87 (0.35) | 0.86 (0.35) | 0.86 (0.39) | <0.0001 |
| EPA^6^, g | 0.064 (0.04) | 0.072 (0.04) | 0.076 (0.04) | 0.081 (0.05) | 0.086 (0.05) | <0.0001 |
| DPA^7^, g | 0.026 (0.01) | 0.029 (0.01) | 0.031 (0.02) | 0.033 (0.02) | 0.036 (0.02) | <0.0001 |
| DHA^8^, g | 0.160 (0.10) | 0.179 (0.10) | 0.192 (0.10) | 0.204 (0.12) | 0.219 (0.12) | <0.0001 |
| Tot n-3 fatty acids^9^, g | 1.17 (0.4) | 1.19 (0.4) | 1.17 (0.4) | 1.18 (0.4) | 1.20 (0.5) | 0.0261 |
| Alcohol | 3.04 (3.0) | 3.74 (3.4) | 4.19 (3.7) | 4.69 (4.2) | 5.24 (4.4) | <0.0001 |
| ***Vitamins*** | | | | | | |
| Vitamin A, RE | 574 (412) | 597 (390) | 625 (419) | 647 (438) | 646 (442) | <0.0001 |
| Vitamin D, µg | 3.30 (1.2) | 3.51 (1.2) | 3.69 (1.2) | 3.84 (1.3) | 3.96 (1.4) | <0.0001 |
| Vitamin E, mg | 4.45 (0.9) | 4.55 (1.0) | 4.53 (0.9) | 4.53 (0.9) | 4.68 (1.0) | <0.0001 |
| Thiamin, mg | 0.67 (0.1) | 0.66 (0.1) | 0.67 (0.1) | 0.67 (0.1) | 0.69 (0.1) | <0.0001 |
| Riboflavin, mg | 1.05 (0.5) | 1.05 (0.4) | 1.06 (0.4) | 1.07 (0.4) | 1.07 (0.3) | 0.0088 |
| Vitamin C, mg | 37.4 (17) | 46.1 (21) | 48.7 (22) | 51.2 (23) | 58.1 (25) | <0.0001 |
| Niacin, NE | 17.4 (2.4) | 18.1 (2.2) | 18.6 (2.2) | 19.3 (2.3) | 20.5 (2.8) | <0.0001 |
| Vitamin B6, mg | 0.97 (0.2) | 1.02 (0.2) | 1.02 (0.2) | 1.04 (0.2) | 1.08 (0.2) | <0.0001 |
| Folate, µg | 149 (31) | 154 (33) | 154 (31) | 155 (33) | 159 (37) | <0.0001 |
| Vitamin B12, µg | 3.25 (1.7) | 3.54 (1.6) | 3.84 (1.7) | 4.10 (1.7) | 4.28 (1.8) | <0.0001 |
| ***Minerals*** | | | | | | |
| Iron, mg | 6.11 (1.2) | 6.12 (1.2) | 6.11 (1.2) | 6.14 (1.2) | 6.36 (1.3) | <0.0001 |
| Zinc, mg | 6.00 (0.7) | 6.11 (0.7) | 6.29 (0.72) | 6.50 (0.8) | 6.80 (0.9) | <0.0001 |
| Calcium, mg | 531 (154) | 568 (172) | 602 (193) | 628 (209) | 619 (199) | <0.0001 |
| Phosphorus, mg | 825 (108) | 838 (113) | 854 (125) | 872 (139) | 877 (135) | <0.0001 |
| Potassium, mg | 2148 (956) | 2147 (844) | 2057 (640) | 2034 (605) | 2090 (574) | <0.0001 |
| Magnesium, mg | 222 (52) | 218 (49) | 210 (39) | 208 (38) | 210 (36) | <0.0001 |
| Selenium, µg | 19.6 (4.4) | 21.3 (4.4) | 22.9 (4.7) | 24.3 (5.2) | 25.9 (5.9) | <0.0001 |
| Sodium, mg | 1397 (168) | 1415 (180) | 1450 (174) | 1498 (191) | 1566 (214) | <0.0001 |
| ^1^SFA ^2^MUFA=Monounsaturated fatty acids. ^3^PUFA=Polyunsaturated fatty acids. ^4^LA= Linoleic acid. ^5^ALA=**α-linolenic acid. ^6^EPA=** Eicosapentaenoic acid. ^7^DPA=Docosapentaenoic acid. ^8^DHA=Docosahexaenoic acid. ^9^Total intake of ALA, EPA, DPA and DHA. | | | | | | |

**Figure S1. Impact of individual environmental indicators in relation to aggregated environmental score (total population).**
